# Hypochondriasis as Dependent on Chronic Gastritis and Enteritis

**Published:** 1829-07-01

**Authors:** 


HYPOCHONDRIASIS AS DEPENDENT ON CHRONIC INFLAMMATION OF THE STOMACH AND BOWELS. By M. DELORMEL, M.D.

The author of this paper, which is published in a recent number of the Archives, has laboured to prove that melancholia and hypochondriasis are but symptoms or phenomena of gastro-enteritis chronica. The difficulty of distinguishing between irritation and inflammation, and the frequent combination of these two states, give considerable colour, and an air of probability to M. Delormel's doctrine, which is opposed to that of some distinguished continental physisians, who refer hypochondriasis directly to the brain. Here again we are puzzled between primary and secondary affections. If the mental energies become impaired or deranged, it is natural to conclude that the material organ of mind is the seat of the disorder. But this disorder is very often an effect of another disorder seated at a considerable distance. In pregnancy and in some uterine diseases, vomiting occurs. It cannot be doubted that the vomiting is produced by disorder of the stomach —but the disorder of the stomach is caused by disorder of the uterus. The same reasoning will apply to hypochondriasis. Irritation of the stomach will pervert the judgment and the imagination. The *immediate* cause of these perversions is, unquestionably, in the brain; but the primary cause may be in the stomach. And even when moral causes act on the sentient system, and thus disturb the intellectual functions, we do not often find the phenomena of hypochondriasis induced till the digestive apparatus has suffered, and has begun to react on the sensorial energies. It is probable then, that, in a practical point of view, our remedial measures will be most advantageously directed to the chylopoietic organs;—since it is through them that we shall be best able to influence the sensorial functions. The following case will furnish food for reflection.

In the month of April our author was called to Madame Chatelain, at the Chateau D'Armanvillers, who, according to the physician in attendance, was "melancholic, hypochondriacal, and insane." She had made several attempts at suicide, and was carefully guarded. She had been bled, purged, and well dosed with antispasmodics; but to no purpose. Dr. Delormel examined for himself, and came to conclusions very different from those of the ordinary medical attendant. The lady was 37 years of age, of very nervo-sanguineous temperament—active in body, and most amiable in her dispositions. For more than two years she had complained of burning heat, at times, in her stomach and bowels—the digestion was painful —and constipation was habitual. The catamenia were irregular and scanty— emaciation progressive—symptoms of melancholia and hypochondriasis well marked. Madame C. now declined all her usual avocations, and kept constantly on her couch. She could not bear to see her husband or children, to whom she was much attached when in health. Her ruling passion was solitude—and the predominant idea was the conviction of approaching death. The lips and tongue were red, with whitish fur on the middle of the latter—the chest sounded well, although she had had cough for three months—the epigastrium and hypochondria were extremely tender on pressure—pulse small and hard—evening fever—disgust for every kind of food—considerable thirst—pupils very dilated—nose pinched in—eyes hollow. She had missed one period of menstruation. The reunion of all these symptoms left no doubt in our author's mind of the existence of chronic gastro-enteritis. The treatment need not be detailed. We shall only observe that, in the course of five days, 80 leeches were applied to the epigastric and hypochondriac regions—that warm hip-baths, and warm fomentations to the abdomen were employed—that the patient was kept at first on sugar and water, and gradually allowed barley water and similar slops, previously to more substantial alimentation. In less than a month Madam Chatelain had lost her gloomy ideas and her fear of death—and these were succeeded by good spirits and rational ideas. In two months this interesting patient was able to take substantial nourishment, and was restored to perfect health.

We have no doubt that the gloominess of mind, in this case, was dependent on gastric affection; but we are inclined to believe that the starvation alone would have produced the desired effect, without such a number of leeches. We are by no means convinced that the disease was gastro-enterite. A highly *irritable* state of the gastric and intestinal mucous membrane will produce all the phenomena which the above patient exhibited—and these phenomena will often be removed by great abstinence at first, and a very gradual return to substantial food afterwards.

---

